# Exploring pharmacological options for adolescent depression: a preclinical evaluation with a sex perspective

**DOI:** 10.1038/s41398-022-01994-y

**Published:** 2022-06-01

**Authors:** Sandra Ledesma-Corvi, Elena Hernández-Hernández, M. Julia García-Fuster

**Affiliations:** 1 IUNICS, University of the Balearic Islands, Palma, Spain; 2 Health Research Institute of the Balearic Islands (IdISBa), Palma, Spain; 3 Present Address: Department of Pharmacology, University of the Basque Country (EHU/UPV), Leioa, Spain

**Keywords:** Depression, Pharmacology

## Abstract

There is an urgent need for developing novel pharmacological treatment options for adolescent depression, and to ensure an optimal translational outcome to the clinic, sex should be included as a biological variable in preclinical studies. In this context, the present study compared the antidepressant-like potential of ketamine and cannabidiol, with the clinical standard fluoxetine, in adolescent rats exposed to maternal deprivation (as a model of early-life stress), while including a sex perspective. Moreover, changes in drug efficacy over time were evaluated by re-exposing rats to the same dose regimens during adulthood. Antidepressant-like responses were scored through a battery of distinctive tests (forced-swim, novelty-suppressed feeding, and sucrose preference) across time. The main results proved an antidepressant-like potential for ketamine and cannabidiol in adolescent rats, although their efficacy was dependent on sex and prior stress exposure, as well as on treatment length and the behavioral feature analyzed. In general, while all tested antidepressants in male rats improved certain affective-like features, female rats were mainly unresponsive to the treatments performed (except for certain benefits induced by ketamine), demonstrating the need for further characterizing proper treatments for this particular sex. Moreover, when rats were re-exposed in adulthood to the same drug regimens as in adolescence, a drop in efficacy was observed. These findings may have translational ramifications in that ketamine or cannabidiol could be moved forward as antidepressants for the adolescent depressed population, but not before further characterizing their potential long-term safety and/or beneficial vs. harmful effects for both sexes.

## Introduction

Major depression affects around 5–6% of adolescents (13–18 years old; [1]), and its treatment is a major cause of concern since, other than a psychological approach (including internet-based platforms), only fluoxetine is recommended as a pharmacological option. The reason behind this lack of options for this age population relies on the fact that the intrinsic mechanisms mediating adolescent depression seem to vary from those in adulthood, since the brain is still in development, and pharmacological treatments often fail to respond, show lower efficacy (e.g., [2–5]), and/or could even induce a harmful response (i.e., increased suicide ideation; [6]). Moreover, the underlying sex differences in relation to puberty and depression [7, 8] (i.e., common rates during early adolescence [9–11], but increased propensity at later ages for females [12–14]), together with the differences in efficacy reported for certain antidepressants with sex (see [15, 16]), as well as the lack of preclinical studies performed in females [17], especially for the adolescent population, ensures a poor translational outcome to the clinic.

The goal of this study was therefore to characterize novel pharmacological antidepressants for adolescence using a rodent model of early-life stress (e.g., [18–20]) and while including sex as a biological variable [21, 17, 8]. In this context, ketamine, an NMDA receptor antagonist, was recently approved by the FDA for treatment-resistant depression and suicidality in adults [22], and while its use in younger populations is currently limited to anesthetic and analgesic purposes [23], an increasing number of clinical reports [24–26] suggested a potential use for this drug in adolescent depression, although special caution is still needed in terms of safety and long-term potential concerns in addiction-liability [27, 28]. In this context, recent studies are centered in evaluating the differential effects of ketamine as influenced by sex (some studies showed improved responses in rodents for females [29–32] or males [33], while others even showed no sex differences in humans [34, 35]), by age [36, 25] and prior stress exposure (e.g., [32, 36–39]), as well as the potential dose-related molecular actions behind these disparities [40–43]. Another good candidate to be further explored as a potential antidepressant for adolescent depression is cannabidiol [44–46], a non-psychotomimetic active compound extracted from the plant *Cannabis Sativa*. Prior preclinical studies in rodents have also proven sex-, age- and stress-related antidepressant-like effects induced by cannabidiol [47, 4, 48, 49], without clear potential harmful behavioral effects [50].

Within this framework, ketamine and cannabidiol emerged as possible novel pharmacological options for adolescent depression and were evaluated in the present study in comparison with the standard fluoxetine [51–56]) in adolescent rats exposed to early life stress (i.e., maternal separation as a potential triggering factor for several mood disorders [57, 58]), while including sex as a biological variable. Antidepressant-like responses were assessed through a battery of distinctive tests (forced-swim, novelty-suppressed feeding, sucrose preference). Moreover, during adulthood, changes in drug efficacy were scored by re-exposing rats to the same dose regimens.

## Materials and methods

### Early-life conditions

**Fig. 1:**
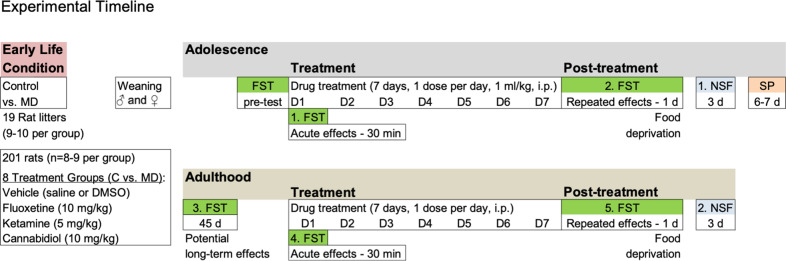
Experimental timeline. A total of 201 rats were exposed in early-life to maternal deprivation (MD) or control conditions, followed by a battery of behavioral tests (FST forced-swim, NSF novelty-suppressed feeding, SP sucrose preference) in mid/late-adolescence and adulthood during and/or following drug treatment (vehicle, ketamine, cannabidiol, and fluoxetine). D day of treatment, d day post-treatment.

All animal experimental procedures were approved by Local Bioethical Committees (University of the Balearic Islands and Conselleria Medi Ambient, Agricultura i Pesca, Direcció General Agricultura i Ramaderia, Govern de les Illes Balears), in accordance to the ARRIVE guidelines [59] and the EU Directive 2010/63/EU. The present study included 3 cohorts of experiments performed over time in 19 Sprague-Dawley litters bred in the animal facility at the University of the Balearic Islands. Whole litters were either exposed to maternal deprivation (10 litters, *n* = 100 pups) or used as naïve controls (9 litters, *n* = 101 pups). Pups exposed to maternal separation remained alone in their home cage with no nutritional supplements during a single 24 h episode from PND 9 to PND 10 while their mothers rested in adjacent cages as previously described [3, 48]. All pups, including those from control groups, were weighted before and after the procedure. On PND 9, their average weight was 16.88 ± 0.26 g. However, on PND 10, while pups from the control litters showed the expected daily increase in body weight (+1.58 g, ***p < 0.001 vs. PND 9), the ones exposed to maternal deprivation showed a significant drop in weight (−0.73 g, ***p < 0.001 vs. PND 9; data not shown in graphs). Rats were separated at weaning in standard cages (2–4 rats/cage) by sex and early-life condition (male control, *n* = 52, female control, *n* = 49, male maternal deprivation, *n* = 51, female maternal deprivation, *n* = 49; Fig. 1), and housed with unlimited access to a standard diet and water in controlled environmental conditions (22 °C, 70% of humidity, and a 12:12 h light/dark cycle, lights on at 8 h). The specific stages of the estrous cycle were not monitored throughout the course of procedures, since the cyclicity was not part of the research question (see [8]), and the effect of the estrous cycle on the forced-swim test baseline performance of naturally cycling females was reported to be small (reviewed by [60]). Yet, stages were evaluated at the time of sacrificing the animals, presenting females with a normal distribution of all phases of the estrous cycle (data not shown), and therefore avoiding the chance of an over-representation of a particular phase.

### Pharmacological drug treatments

Given the high number of experimental groups per cohort in which to run behavior (8 experimental groups: male vs. female, control vs. maternal deprivation, vehicle vs. drug treatment) only one dose was tested per drug, which was selected from prior studies (mainly done in adult male rats, see references below). To do so, randomly allocated rats from each sex and early-life condition (*n* = 8–9 rats per group; see Fig. 1) were treated during a period comprised between mid and late-adolescence (as described in [61]) for 7 consecutive days (1 injection per day, i.p., 1 ml/kg) with vehicle (0.9% NaCl or DMSO), ketamine (Richter Pharma, Austria; dose: 5 mg/kg, selected from [37]), cannabidiol (THC Pharm GmbH, Germany; dose: 10 mg/kg, selected from [4, 48]) or fluoxetine (Grupo Ferrer, Spain; dose: 10 mg/kg, selected from [51, 54]). Later on, rats were re-exposed in adulthood to the same drug regimen they received in adolescence to evaluate potential drops in efficacy over time (see Fig. 1).

### Forced-swim test

This test was originally designed to evaluate acute antidepressant-like activity in rodents [62], and is also used to characterize potential novel therapeutic options for adolescent depression (see some of our prior publications in [3–5]). In the present study, rats were placed in individual cylinders (41 cm high × 32 cm diameter), and filled with water (25 ± 1 °C; up to 25 cm in depth). At the beginning of the pre-test session, each rat was gently placed in the tank and allowed to swim about freely for 15 min, which was followed, 48 h later, by a 5 min test session that was videotaped (see Fig. 1). At the end of each session, rats were dried off with a paper towel and returned to their cage. The test was repeated across time as it was used to evaluate the acute (30 min) and repeated (1-day post-treatment) effects induced by each drug under evaluation during adolescence, the potential long-lasting effects following adolescent exposure, as well as the acute and repeated effects following drug re-exposure in adulthood (see Fig. 1). In later analysis, videos were blindly scored (Behavioral Tracker software, CA, USA) to determine the time each rat spent (s) immobile vs. in active behaviors (climbing or swimming).

### Novelty-suppressed feeding test

This test was first described to assess differences in sensitivity to novelty in an anxiogenic-like environment (e.g., [63, 64]), and is generally used to score chronic antidepressant-like responses (e.g., [65, 48]). To do so, rats were food-deprived prior to testing for 48 h (Fig. 1), since the motivation for food is required. During testing, which was done under housing illumination conditions 3 days post-treatment (both in adolescence and, later on, in adulthood after drug re-exposure), rats were individually placed for 5 min in a square open-field arena (60 cm × 60 cm, and 40 cm in high) with three food pellets in the center (e.g., [48]). Sessions were videotaped and the parameters of video analysis that were evaluated for each rat were latency to center (s), feeding time (s), and distance traveled (cm).

### Sucrose preference test

This test is based on the fact that rodents, when given the chance, prefer a sweet solution (e.g., 1% sucrose) over water (two-bottle choice test), representing an indicator of their hedonic-like response [66], and used as an indicator of anhedonia (a core symptom of depression). Before sucrose presentation, rats were trained to drink from two water bottles placed on each side of the housing cage (day 5 post-treatment), to ensure they drink equally from both sides. Then, sucrose intake (g/kg) and sucrose preference (%) were scored per cage (groups of 2–4 rats/cage) on days 6 and 7 post-treatment during adolescence (Fig. 1). Briefly, for 48 h, rats were allowed to freely drink from two bottles containing 1% sucrose or water [4, 5], which were placed in alternate positions every day to prevent bias towards any side of the cage. The test was performed in whole cages to avoid the stress of individually housing rats (i.e., social isolation) for extended periods of time (e.g., [67]), since the experimental design lasted several months (see Fig. 1). On day 8 post-treatment rats were presented again with two water bottles to ensure an equal preference for both of them.

### Data analysis and statistics

Data were analyzed with GraphPad Prism, Version 9.3.0 (GraphPad Software, Inc., USA). Results are presented as mean values ± standard errors of the mean (SEM), with symbols representing individual values for each rat, in line with the recommendations for displaying data and statistical results in experimental pharmacology (e.g., [68, 69]). All data generated was evaluated by three-way ANOVAs (independent variables: sex, early-life, and treatment) and/or, when assessing each sex separately, by two-way ANOVAs (independent variables: early-life and treatment). When appropriate, multiple comparisons were performed by Tukey’s test. The level of significance was fixed at *p* ≤ 0.05.

## Results

### Early-life stress as a mild model of negative affect

In an attempt to behaviorally characterize the animal model utilized, as well as the impact of repeating behavioral tests across time, before reporting the effects of drug treatment, the impact of early-life stress was evaluated over time in the cohorts of vehicle-treated rats (a total of *n* = 49 controls and *n* = 49 MD rats; see Supplementary Fig. S1). The progression of affective-like responses through time (adolescence vs. adulthood), both in the forced-swim and novelty-suppressed feeding tests, suggested the absence of significant responses as induced by maternal deprivation for both sexes (see Supplementary Fig. S1). Some differences emerged in test performance with time, such as lower immobility and higher climbing rates in the forced-swim test (i.e., indicative of a more active/escaping behavior, which for female rats was presented as higher swimming rates), and higher latencies to center paired with lower feeding times and distance traveled in the novelty-suppressed feeding test (i.e., indicative of a worse behavioral outcome probably caused by the loss of novelty with test repetition; Supplementary Fig. S1). Interestingly, some basal sex differences also emerged in the novelty-suppressed feeding test, such as an overall lower latency to the center and a higher feeding time and distance traveled for female vs. male rats (see Supplementary Fig. S1).

Remarkably, when also considering treatment in the analysis (three-way ANOVAs with independent variables: sex, early-life, treatment), early-life stress-induced significant changes in negative affect, as observed in the forced-swim and novelty-suppressed feeding tests (see Supplementary Table S1). More importantly, the drugs tested responded in a different way depending on whether rats were previously exposed to early-life stress or not (treatment x early-life condition interactions, see Supplementary Table S1).

### Antidepressant-like effects during adolescence

Given the observed sex and test repetition (or age maturity) differences in effect for all cohorts of vehicle-treated rats (see Supplementary Fig. S1), as well as the sex differences reported when analyzing all data through three-way ANOVAs (see Supplementary Table S1), the effects of drug treatments were reported in control and maternally deprived rats for each sex separately, and analyzed through two-way ANOVAs (independent variables: early-life condition and treatment; see Supplementary Table S2) at each day of testing (Fig. 1).

**Fig. 2:**
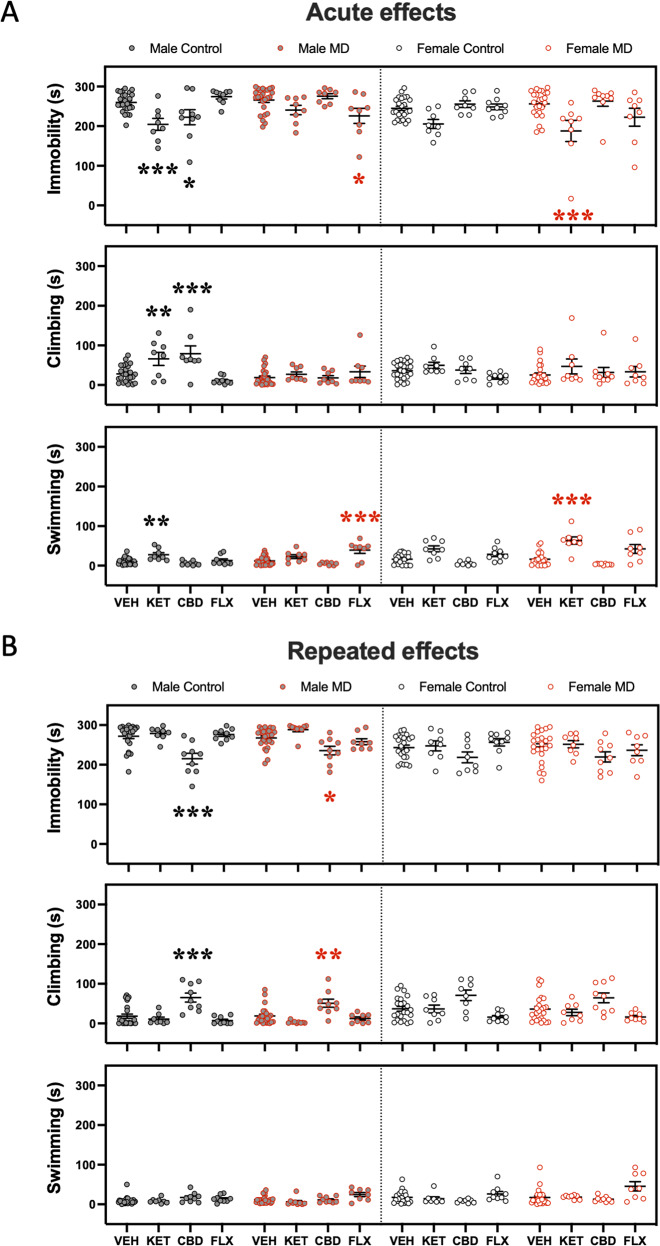
Antidepressant-like effects as measured in the forced swim test (FST) during adolescence. **A** Acute (30 min post-treatment) and **B** repeated (1-day post-treatment) effects exerted by adolescent ketamine, cannabidiol, or fluoxetine exposure in male and female rats (Control or MD) in the FST. Data represent mean ± SEM of the time spent (s) immobile, climbing, or swimming. Individual values are shown for each rat (symbols). Three-way ANOVAs (independent variables: sex, early-life, and treatment) and two-way ANOVAs (independent variables: early-life and treatment) are shown in Supplementary Tables 1 and 2 respectively. ***p < 0.001, **p < 0.01, and *p < 0.05 vs. the appropriate control group (Control or MD) and sex.

Particularly, in male adolescent control rats, both ketamine and cannabidiol-induced an acute antidepressant-like effect as measured in the forced-swim test 30 min post a single injection (i.e., decreased immobility); while ketamine increased active behaviors in general (climbing and swimming), cannabidiol exclusively increased climbing behavior (Fig. 2A). These effects were not observed in male rats exposed to maternal deprivation early in life. Contrarily, fluoxetine induced an acute antidepressant-like effect in adolescent male rats but only in those exposed to maternal deprivation (i.e., decreased immobility and increased swimming; Fig. 2A). Moreover, as for the repeated drug treatments, only cannabidiol rendered efficacious in male adolescent rats, both in controls and maternally deprived rats, by proving an antidepressant-like effect as measured in the forced-swim test (i.e., decreased immobility and increased climbing; Fig. 2B). Interestingly, most of the drugs tested during adolescence in the forced-swim test were not effective in female rats (neither acutely nor repeatedly; Fig. 2), except for acute ketamine that exerted an antidepressant-like effect in maternally deprived rats (i.e., decreased immobility and increased swimming; Fig. 2A).

**Fig. 3:**
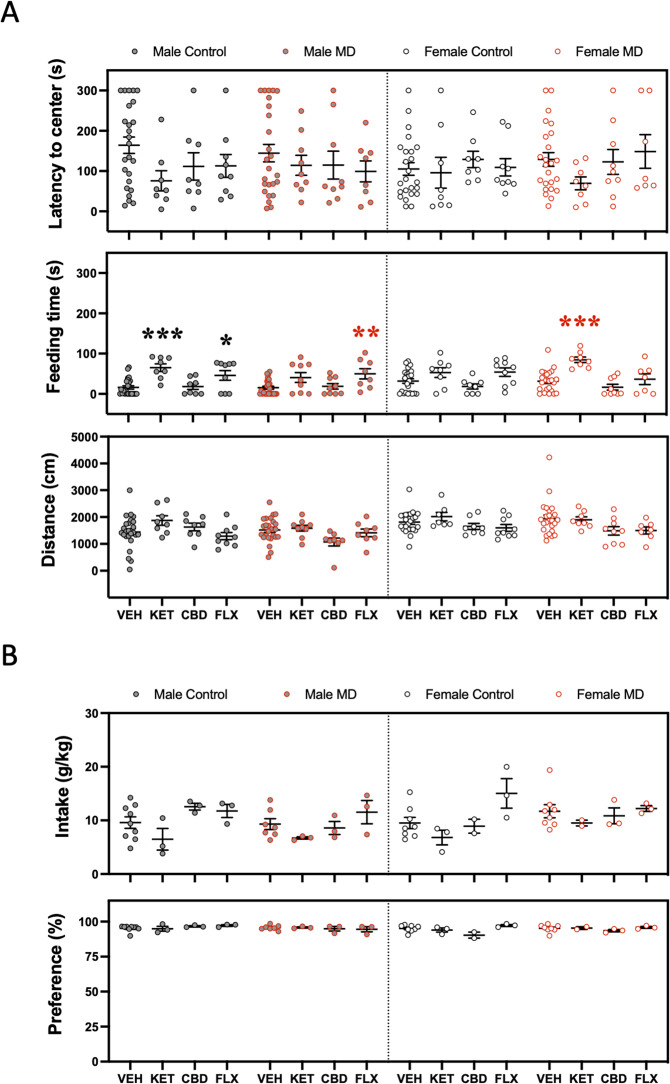
Antidepressant-like effects as measured in the novelty-suppressed feeding test (NSF) or sucrose preference test (SP) during adolescence. **A** Repeated effects (3 days post-treatment) exerted by adolescent ketamine, cannabidiol, or fluoxetine exposure in male and female rats (Control or MD) in the NSF. Data represent mean ± SEM of the latency to center (s), feeding time (s), or distance traveled (cm). **B** Repeated effects (6–7 days post-treatment) exerted by adolescent ketamine, cannabidiol, or fluoxetine exposure in male and female rats (Control or MD) in the SP. Data represent mean ± SEM of the sucrose intake (g/kg) or preference (%). Individual values are shown for each rat (symbols). Three-way ANOVAs (independent variables: sex, early-life, and treatment) and two-way ANOVAs (independent variables: early-life and treatment) are shown in Supplementary Tables 1 and 2 respectively. ***p < 0.001, **p < 0.01 and *p < 0.05 vs. the appropriate control group (Control or MD) and sex.

The novelty-suppressed feeding test was performed during adolescence 3 days post-treatment. The results showed that ketamine increased the time rats spent feeding (i.e., an indicative of an antidepressant- and/or anxiolytic-like effect), but only in male adolescent control rats. Contrarily, no effects were observed for cannabidiol in male adolescent rats. Interestingly, fluoxetine also increased the time control and maternal-deprived adolescent male rats spent feeding. Again, and similarly to the results presented in the forced-swim test, ketamine was the only drug that exerted a putative beneficial effect in females, but only detected in maternally deprived rats (i.e., increased feeding time; Fig. 3A). As the data showed, the changes in feeding time (s) did not seem to be driven by changes in distance traveled (cm) or latency to center (s), since they were not altered by any treatment studied (see Fig. 3A).

Finally, no significant changes were observed when measuring 1% sucrose intake (g/kg) or preference (%) as induced by any of the drugs tested in adolescence, and as measured in the sucrose preference test in male and female rats 6–7 days post-treatment (Fig. 3B), suggesting the importance of utilizing complementary tests for scoring different aspect of affective-like behavior. Moreover, no changes were observed in daily water consumption among groups (data not shown).

### Antidepressant-like effects during adulthood

**Fig. 4:**
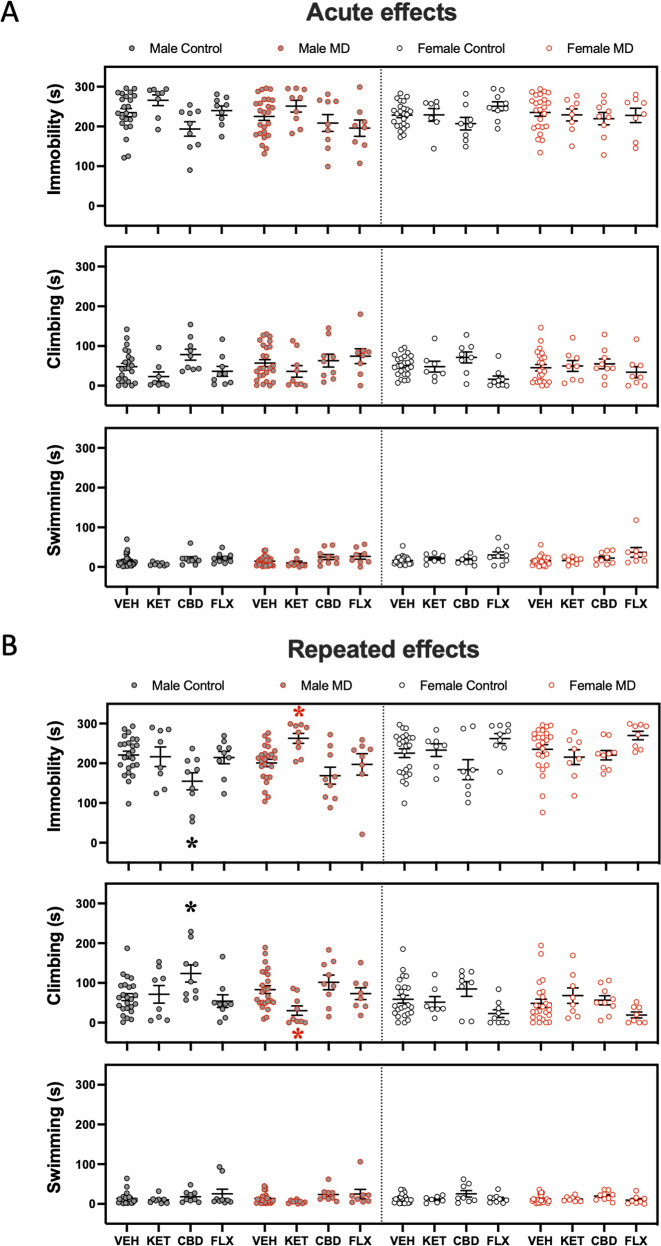
Antidepressant-like effects as measured in the forced swim test (FST) during adulthood. **A** Acute (30 min post-treatment) and **B** repeated (1-day post-treatment) effects exerted by adult ketamine, cannabidiol, or fluoxetine exposure in male and female rats (Control or MD) in the FST. Data represent mean ± SEM of the time spent (s) immobile, climbing, or swimming. Individual values are shown for each rat (symbols). Three-way ANOVAs (independent variables: sex, early-life, and treatment) and two-way ANOVAs (independent variables: early-life and treatment) are shown in Supplementary Tables 1 and 2 respectively. *p < 0.05 vs. the appropriate control group (Control or MD) and sex.

The potential long-term effects of each adolescent treatment were evaluated in the forced swim test 45 days post-treatment. The results showed that neither ketamine, cannabidiol, nor fluoxetine induced persistent changes in immobility, climbing or swimming behaviors (see Supplementary Fig. S2). Then, 5 days later, rats were re-exposed to the same drug regimens as received in adolescence and were tested in the forced swim test (see Fig. 1). None of the drugs evaluated (ketamine, cannabidiol, and fluoxetine) induced an acute antidepressant-like response as measured in the forced-swim test in male and female rats, and independently of the early-life condition experienced (see Fig. 4A). Remarkably, 24 h post-repeated treatments, cannabidiol was the only drug that produced an antidepressant-like effect, observed for male control rats in the forced swim test (i.e., decreased immobility and increased climbing; Fig. 4B), similarly to the results reported in adolescence. On the contrary, repeated ketamine administration increased immobility and reduced climbing in adult male maternal-deprived rats (see Fig. 4B), indicative of a negative impact on affective-like behavior. Finally, female rats were even more unresponsive than in adolescence, since no signs of efficacy were induced by any of these treatments in adulthood (Fig. 4).

**Fig. 5:**
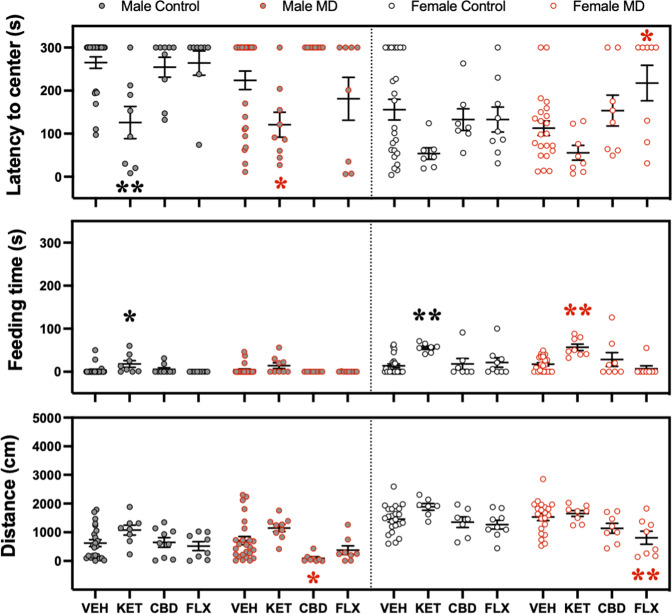
Antidepressant-like effects as measured in the novelty-suppressed feeding test (NSF) during adulthood. Repeated effects (3 days post-treatment) exerted by adult ketamine, cannabidiol, or fluoxetine exposure in male and female rats (Control or MD) in the NSF. Data represent mean ± SEM of the latency to center (s), feeding time (s), or distance traveled (cm). Individual values are shown for each rat (symbols). Three-way ANOVAs (independent variables: sex, early-life, and treatment) and two-way ANOVAs (independent variables: earlylife and treatment) are shown in Supplementary Tables 1 and 2 respectively. **p < 0.01 and *p < 0.05 vs. the appropriate control group (Control or MD) and sex.

In the novelty-suppressed feeding test (as measured 3 days post-treatment in adulthood), ketamine decreased the latency to center (s) both in control and maternally deprived male rats. Moreover, ketamine increased the time rats spent feeding (s) in male controls, as well as in female rats (both control and maternally deprived), indicative of antidepressant- and/or anxiolytic-like responses (Fig. 5). Contrarily, cannabidiol and fluoxetine did not induce any beneficial effects as measured in this test both for male and female rats. In fact, they even seemed to be somehow harmful, since cannabidiol decreased the distance traveled (cm) in maternally deprived male rats, and fluoxetine increased the latency to center (s) and decreased the distance traveled (cm) in female maternal-deprived rats (Fig. 5). Food intake and water consumption were not monitored throughout the procedure so no conclusions could be extracted on the possible orexigenic and/or dipsogenic drug effects that could be confounding the present results.

## Discussion

This behavioral study proved that the antidepressant-like effects induced by ketamine, cannabidiol, or fluoxetine during adolescence were dependent on sex and prior stress exposure, as well as on treatment length and the behavioral dimension analyzed. In general, all three antidepressants improved different aspects of affective-like behavior for male rats, although prior early-life stress altered the outcome in some cases. However, female rats were mainly unresponsive to the treatments performed (except for the observed benefits induced by ketamine), demonstrating the need for further characterizing other doses, and/or finding proper treatments for this particular sex, to ensure optimal translational outcomes. The antidepressant-like effects induced during adolescence did not produce long-term responses, and when rats were re-exposed in adulthood to the same drug regimens as in adolescence, no acute effects were observed. In fact, a repeated drug regimen was needed to induce certain antidepressant-like responses by ketamine or cannabidiol, suggesting a drop in efficacy with age (adulthood vs. adolescence) following drug re-exposure.

The paradigm of early-life stress utilized in this study was mild in terms of inducing basal differences in affective-like behavior (maternally deprived vs. naïve control rats), in line with some prior published literature stating that preclinically, the maternal separation paradigm, used to examine the influence of this form of early-life stress in the first weeks of life, did not always and necessarily produce a depressive-like phenotype (reviewed by [70]). In this context, reproducibility in rodent behavioral research is a topic of great debate and concern and varies for each model depending on the particular experimental conditions. Particularly, we have used this specific paradigm of maternal separation (24 h from PND 9 to 10) in our group for some time now, publishing four independent studies including hundreds of rats [3, 48, 71, 72]. The main conclusion of all of them is that, in our hands, depriving Sprague-Dawley pups 24 h from their mom from PND 9 to PND 10, seems to be a mild procedure in terms of altering affective-like responses, at least with the behavioral tests that we normally utilize (e.g., forced-swim test, novelty-suppressed feeding, and sucrose preference). However, this lack of behavioral impact is accompanied by changes in neurochemical markers in the hippocampus (i.e., increased pro-apoptotic FADD [71]), a brain region particularly responsive to stress, suggesting a negative impact induced by this procedure. Moreover, the combination of maternal separation with a different stressor (i.e., drug administration during adolescence) induced negative affect [71, 72], in line with the literature reporting the “two-hit” hypothesis in the development of neuropsychiatric illnesses (a combination of two or more major disruptions are needed at specific time points during development). Moreover, it is important to remember that the forced-swim test is a good test to measure antidepressant-like responses, but not necessarily to phenotype a depressive-like response (reviewed by [73]). Interestingly, the fact that the model induced sex differences in response to this prior stressor, but also changes in treatment responses (as observed by the significant interactions reported), suggested an impact of the procedure and supported its suitable use for the objective of the study. In fact, we selected maternal deprivation as a simple model (with easy logistics) in which to perform the first drug screening in adolescence following an early-life stressor. To model a more robust depressive-like phenotype we would have needed other preclinical approaches that include more aggressive and/or prolonged procedures that might have even interfered with the timing of the adolescent treatment (e.g., [19]). Therefore, early-life stress modeled with maternal separation, although failed to show baseline changes in affective-like behavior with the tests performed (as previously described in [71, 72]), proved differences in the effectiveness of the selected drug treatments, both in adolescence and adulthood, presenting itself as a good platform in which to explore novel antidepressant options for adolescence with a sex perspective.

In this regard, the present results demonstrated a great potential for ketamine as an antidepressant for adolescence, since an acute dose improved immobility in the forced swim test, and a repeated treatment increased feeding time in the novelty-suppressed feeding, both indicators of positive preclinical outcomes. However, when ketamine was re-exposed in adulthood to the same rats that received the drug in adolescence, a repeated paradigm was needed to observe the antidepressant-like response, since no acute effects were detected. Interestingly, although these effects across time were observed for both sexes, they were defined by early-life stress exposure, since ketamine showed a parallel efficacy in control male rats and maternally deprived female rats. No beneficial effects were present for maternally deprived male rats (even a deleterious effect was induced by repeated ketamine in adulthood) as well as for control female rats, suggesting sex- and stress-related interactions in the observed drug responses. In this regard, prior studies in the literature have already reported some sex- [29–33] and stress-related (e.g., [32, 36–39]) differential responses for ketamine, however, most of these earlier studies were performed in adult rodents (as opposed to adolescent), mainly in mice, and utilized other procedures to model stress-related conditions (not maternal separation). For example, a recent study reported that chronic psychological stress could also interact with ketamine to modulate its effects since it was capable of inducing an antidepressant-like effect 24 h post-injection (i.e., decreased immobility and increased swimming in the forced-swim test) in mice exposed to unpredictable chronic stress, while it induced the opposite effects in unstressed mice [38]. Moreover, when evaluating the impact that sex induced on the sustained effects of ketamine on resilience to chronic stress, a recent report in adult mice showed that while ketamine prevented chronic stress-induced changes in affective-like behaviors in males, it did not protect females [33]. Furthermore, the drop in ketamine’s efficacy after re-exposure in adulthood could be mediated by the emerging differences driven by age [36, 25] and/or be dose-related [40–43]. Our data suggested a great antidepressant-like potential for ketamine in adolescence, while also demonstrated that maternal deprivation interacted with ketamine treatment to modulate its effects in female rats. Therefore, future studies should center on ascertaining the potential mechanisms behind ketamine’s actions in relation to the differential effects observed by sex- and stress- (as reviewed in [40, 41]), and incorporating other doses/regimens of exposure that might still provide efficacy in adulthood, since most probably, adolescent patients will need to be further medicated at later ages. Still, caution should be taken with these data since the use of ketamine during adolescence might induce dissociative effects and long-term drug abuse problems (although low doses seem to be safe; e.g., [36, 37]), being these side effects a possible limitation to its translational use in the clinic.

In regards to the potential antidepressant-like effects of cannabidiol in adolescence, the results showed both acute and repeated efficacies as observed in the forced-swim test by decreasing immobility and increasing climbing rates, but only for male control rats. Male rats exposed to maternal deprivation needed a repeated regimen exposure for cannabidiol to exert its antidepressant-like actions in adolescence. Similarly, when cannabidiol was re-exposed to rats in adulthood, a repeated paradigm was necessary to observe the expected antidepressant-like effects in controls, while the response was no longer detected in maternally deprived male rats. Prior preclinical studies have also reported age- and stress-related antidepressant-like effects induced by cannabidiol in male rodents [4, 47, 48]. In our case, to select the dose and length of the drug treatment, we relied on published literature on the topic, which varied from dose regimens from 7 and up to 21 days of treatment (e.g., [49]). However, we decided to start with the shortest paradigm of 7 days of cannabidiol administration at the dose of 10 mg/kg, since this was the dose and length that showed an antidepressant-like effect in our previous study in male adolescent rats (see [4]). We acknowledge that although it would have been interesting to assess how other doses and/or even longer treatments could affect the results, we first decided to check the potential sex- and/or stress-related effects of a dose known to induce a response in adolescent naïve male rats. Our results validated our prior study [4], and extended it by including cannabidiol’s acute antidepressant-like potential in male rats, and its lack of efficacy in maternally deprived rats. Moreover, the present study also evaluated cannabidiol’s potential in female rats, but unfortunately, no antidepressant-like responses were observed. Similarly, acute cannabidiol-induced an antidepressant-like effect in male but not in female mice [49]. Therefore, the results proved a great potential for cannabidiol (at the specific conditions tested) as a potential antidepressant for male adolescent and adult rodents in line with prior literature ([4, 48] reviewed in [44–46]), while suggesting that other doses/regimens of exposure will need to be tested to find effective treatments for females.

Finally, fluoxetine, which was selected as the clinical standard, only induced an antidepressant-like effect in adolescent male rats exposed to maternal deprivation (serving as a positive control only in this scenario), as observed acutely in the forced-swim test (i.e., decreased immobility and increased swimming) and repeatedly in the novelty-suppressed feeding test (i.e., increased feeding time). No efficacy was detected following re-exposure in adulthood, but most importantly it rendered inefficacious for female rats at any age and condition tested. However, some prior studies have proven fluoxetine to work during adolescence not only in males [51, 54], but also in female rodents [53, 54], and/or to render sex- and age-related responses (e.g., [56]). In any case, the present results are really relevant since although fluoxetine is the clinical standard for adolescent depression, in our study only worked for male rats, suggesting the need for a deeper characterization for female rodents, since other doses and/or paradigms will need to be adjusted for optimal therapeutical responses and clinical translation for this particular sex.

Overall, these findings may have translational ramifications in that ketamine or cannabidiol could be moved forward as antidepressants for the adolescent depressed population, but not before further characterizing their potential long-term safety and/or beneficial vs. harmful effects. Interestingly, drugs responded differently based on prior stress exposure during early-life. Moreover, these results reinforce the need to include female rats in all preclinical studies, since the expected efficacy of the tested antidepressants was diminished or even inexistent for females (except for ketamine). The differential regulation of these antidepressants by sex deserves a broadened characterization in terms of what drives the behavioral differences and to figure out the potential molecular correlates involved in those actions. These studies are currently ongoing since they will not only include the brains of the present rats sacrificed after the behavioral phenotyping (which will only provide a “photo-finish” of the brain), but also new experiments with rats treated with the same drug paradigms and sacrificed at the specific time points in which behavior differed by sex and/or prior stress condition, so the time-course of the effects could be ascertained. So far, if one were to select one drug to move it forward to the clinic, and although ketamine is already approved by the FDA for resistant depression in adulthood and seems to work for both sexes in adolescence, it has special surveillance for addictive and/or harmful potential, making it not an ideal drug candidate to be administered in our young population. On the other hand, cannabidiol has proven a great potential in adolescence and is already in the clinic for certain types of resistant epilepsy in children, providing some data in terms of its long-term safety. Therefore, once a better understanding is gained on its potential use at this early-life age, and further characterization is done in terms of efficacy for females, we should encourage starting clinical trials in human adolescent subjects suffering from major depression that are not responding to psychotherapy and/or the only recommended drug treatment available for this age-range (i.e., fluoxetine).

## Supplementary information

Supplemental Materials

## References

[1] Costello EJ, Ekranli A, Angold A (2006). Is there an epidemic of child or adolescent depression?. J Child Psychol Psychiatry.

[2] Bylund DB, Reed AL (2007). Childhood and adolescent depression: why do children and adults respond differently to antidepressant drugs?. Neurochem Int.

[3] García-Cabrerizo R, Ledesma-Corvi S, Bis-Humbert C, García-Fuster MJ (2020). Sex differences in the antidepressant-like potential of repeated electroconvulsive seizures in adolescent and adult rats: regulation of the early stages of hippocampal neurogenesis. Eur Neuropsychopharmacol.

[4] Bis-Humbert C, García-Cabrerizo R, García-Fuster MJ (2020). Decreased sensitivity in adolescent versus adult rats to the antidepressant-like effects of cannabidiol. Psychopharmacology..

[5] Bis-Humbert C, García-Cabrerizo R, García-Fuster MJ (2021). Dose-dependent opposite effects of nortriptyline on affective-like behavior in adolescent rats: comparison with adult rats. Eur J Pharm.

[6] Cipriani A, Zhou X, Del Giovane C, Hetrick SE, Qin B, Whittington C (2016). Comparative efficacy and tolerability of antidepressants for major depressive disorder in children and adolescents: a network meta-analysis. Lancet..

[7] Beltz AM (2018). Gendered mechanisms underlie the relation between pubertal timing and adult depressive symptoms. J Adolesc Health.

[8] Beltz AM, Beery AK, Becker JB (2019). Analysis of sex differences in pre-clinical and clinical data sets. Neuropsychopharmacology..

[9] Costello EJ, Pine DS, Hammen C, March JS, Plotsky PM, Weissman MM (2002). Development and natural history of mood disorders. Biol Psychiatry.

[10] Coryell W, Solomon D, Leon A, Fiedorowicz JG, Schettler P, Judd L (2009). Does major depressive disorder change with age?. Psychol Med.

[11] Hofmann SG (2020). The age of depression and its treatments. JAMA Psychiatry..

[12] Kessler RC (2003). Epidemiology of women and depression. J Affect Disord..

[13] Marcus SM, Young EA, Kerber KB, Kornstein S, Farabaugh AH, Mitchell J (2005). Gender differences in depression: findings from the STAR*D study. J Affect Disord.

[14] Eid RS, Gobinath AR, Galea LAM (2019). Sex differences in depression: insights from clinical and preclinical studies. Prog Neurobiol.

[15] LeGates TA, Kvarta MD, Thompson SM (2019). Sex differences in antidepressant efficacy. Neuropsychopharmacology..

[16] Herzog DP, Wegener G, Lieb K, Müller MB, Treccani G (2019). Decoding the mechanism of action of rapid-acting antidepressant treatment strategies: does gender matter?. Int J Mol Sci..

[17] Docherty JR, Stanford SC, Panattieri RA, Alexander SP, Cirino G, George CH (2019). Sex: a change in our guidelines to authors to ensure that this is no longer an ignored experimental variable. Br J Pharm.

[18] Ramaker MJ, Dulawa SC (2017). Identifying fast-onset antidepressants using rodent models. Mol Psychiatry.

[19] Gururajan A, Reif A, Cryan JF, Slattery DA (2019). The future of rodent models in depression research. Nat Rev Neurosci.

[20] Bale TL, Abel T, Akil H, Carlezon W, Moghaddam B, Nestler EJ (2019). The critical importance of basic animal research for neuropsychiatric disorders. Neuropsychopharmacology..

[21] Miller LR, Marks C, Becker JB, Hurn PD, Chen WJ, Woodruff T (2017). Considering sex as a biological variable in preclinical research. FASEB J.

[22] McIntyre RS, Rosenblat JD, Nemeroff CB, Sanacora G, Murrough JW, Berk M (2021). Synthesizing the evidence for ketamine and esketamine in treatment-resistant depression: an international expert opinion on the available evidence and implementation. Am J Psychiatry.

[23] Li L, Vlisides PE (2016). Ketamine: 50 years of modulating the mind. Front Hum Neurosci.

[24] Kim S, Rush BS, Rice TR (2021). A systematic review of therapeutic ketamine use in children and adolescents with treatment-resistant mood disorders. Eur Child Adolesc Psychiatry.

[25] Di Vincenzo JD, Siegel A, Lipsitz O, Ho R, Teopiz KM, Ng J (2021). The effectiveness, safety and tolerability of ketamine for depression in adolescent and older adults: a systematic review. J Psychiatr Res.

[26] Dwyer JB, Landeros-Weisenberger A, Johnson JA, Londono TA, Flores JM, Nasir M (2021). Efficacy of intravenous ketamine in adolescent treatment-resistant depression: a randomized midazolam-controlled trial. Am J Psychiatry.

[27] Sanacora G, Heimer H, Hartman D, Mathew SJ, Frye M, Nemeroff C (2017). Balancing the promise and risks of ketamine treatment for mood disorders. Neuropsychopharmacology..

[28] Ochs-Ross R, Wajs E, Daly EJ, Zhang Y, Lane R, Lim P, et al. Comparison of long-term efficacy and safety of esketamine nasal spray plus oral antidepressant in younger versus older patients with treatment resistant depression: post-hoc analysis of SUSTAIN-2, a long-term open-label phase 3 safety and efficacy study. Am J Geriatr Psychiatry. 2022;30:541–56 10.1016/j.jagp.2021.09.014. 10.1016/j.jagp.2021.09.014 34750057

[29] Carrier N, Kabbaj M (2013). Sex differences in the antidepressant-like effects of ketamine. Neuropharmacology..

[30] Franceschelli A, Sens J, Herchick S, Thelen C, Pitychoutis PM (2015). Sex differences in the rapid and the sustained antidepressant-like effects of ketamine in stress-naïve and “depressed” mice exposed to chronic mild stress. Neuroscience..

[31] Sarkar A, Kabbaj M (2016). Sex differences in effects of ketamine on behavior, spine density, and synaptic proteins in socially isolated rats. Biol Psychiatry.

[32] Fitzgerald PJH, Kounelis-Wuillaume SK, Gheidi A, Morrow JD, Spencer-Segal JL, Watson BO. Sex- and stress-dependent effects of a single injection of ketamine on open field and forced swim behavior. Stress. 2021;24:857–65 10.1080/10253890.2021.1871600. 10.1080/10253890.2021.1871600 PMC8325703 33517825

[33] Okine T, Shepard R, Lemanski E, Coutellier L (2020). Sex differences in the sustained effects of ketamine on resilience to chronic stress. Front Behav Neurosci.

[34] Freeman MP, Papakostas GI, Hoeppner B, Mazzone E, Judge H, Cusin C (2019). Sex differences in response to ketamine as a rapidly acting intervention for treatment resistant depression. J Psychiatr Res.

[35] Jones RR, Freeman MP, Kornstein SG, Cooper K, Daly EJ, Canuso CM, et al. Efficacy and safety of esketamine nasal spray by sex in patients with treatment-resistant depression: findings from short-term randomized, controlled trials. Arch Womens Ment Health. 2022;25:313–26 10.1007/s00737-021-01185-6. 10.1007/s00737-021-01185-6 PMC8921149 34973081

[36] Strong CE, Kabbaj M (2018). On the safety of repeated ketamine infusions for the treatment of depression: effects of sex and developmental periods. Neurobiol Stress.

[37] Parise EM, Alcantara LF, Warren BL, Wright KN, Hadad R, Sial OK (2013). Repeated ketamine exposure induces an enduring resilient phenotype in adolescent and adult rats. Biol Psychiatry.

[38] Fitzgerald PJH, Yen JY, Watson BO (2019). Stress-sensitive antidepressant-like effects of ketamine in the mouse forced swim test. PLoS ONE.

[39] Logue J, Schoepfer K, Brea Guerrero A, Zhou Y, Kabbaj M (2022). Sex-specific effects of social isolation stress and ketamine on hippocampal plasticity. Neurosci Lett.

[40] Matveychuk D, Thomas RK, Swainson J, Khullar A, MacKay MA, Baker GB (2020). Ketamine as an antidepressant: overview of its mechanisms of action and potential predictive biomarkers. Ther Adv Psychopharmacol.

[41] Lavender E, Hirasawa-Fujita M, Domino EF (2020). Ketamine’s dose related multiple mechanisms of actions: Dissociative anesthetic to rapid antidepressant. Behav Brain Res.

[42] Parise EM, Parise LF, Sial OK, Cardona-Acosta AM, Gyles TM, Juarez B (2021). The resilient phenotype induced by prophylactic ketamine exposure during adolescence is mediated by the ventral tegmental are-nucleus accumbens pathway. Biol Psychiatry.

[43] Weston RG, Fitzgerald PJ, Watson BO (2021). Repeated dosing of ketamine in the forced swim test: are multiple shots better than one?. Front Psychiatry.

[44] García-Gutiérrez MS, Navarrete F, Gasparyan A, Austrich-Olivares A, Sala F, Manzanares J (2020). Cannabidiol: a potential new alternative for the treatment of anxiety, depression, and psychotic disorders. Biomolecules..

[45] Stanciu CN, Brunette MF, Teja N, Budney AJ (2021). Evidence for use of cannabinoids in mood disorders, anxiety disorders, and PTSD: a systematic review. Psychiatr Serv.

[46] Gonzalez-Cuevas G, Garcia-Gutierrez MS, Navarrete F, de Guglielmo G, Manzanares J (2021). Editorial: cannabidiol treatment in neurotherapeutic interventions. Front Pharm.

[47] Shbiro L, Hen-Shoval D, Hazut N, Rapps K, Dar S, Zalsman G (2019). Effects of cannabidiol in males and females in two different rat models of depression. Physiol Behav.

[48] Bis-Humbert C, García-Cabrerizo R, García-Fuster MJ (2021). Antidepressant-like effects of cannabidiol in a rat model of early-life stress with or without adolescent cocaine exposure. Pharm Rep..

[49] Silote GP, Gatto MC, Eskelund A, Guimaraes FS, Wegener G, Joca SRL (2021). Strain-, sex-, and time-dependent antidepressant-like effects of cannabidiol. Pharmaceuticals..

[50] Kaplan JS, Wagner JK, Reid K, McGuinness F, Arvila S, Brooks M (2021). Cannabidiol exposure during the mouse adolescent period is without harmful behavioral effects on locomotor activity, anxiety, and spatial memory. Front Behav Neurosci.

[51] Iñiguez SD, Warren BL, Bolaños-Guzmán CA (2010). Short- and long-term functional consequences of fluoxetine exposure during adolescence in male rats. Biol Psychiatry.

[52] Freund N, Thompson BS, Denormandie J, Vaccarro K, Andersen SL (2013). Windows of vulnerability: maternal separation, age, and fluoxetine on adolescent depressive-like behavior in rats. Neuroscience..

[53] Yoo SB, Kim BT, Kim JY, Ryu V, Kang DW, Lee JH (2013). Adolescence fluoxetine increases serotonergic activity in the raphe-hippocampus axis and improves depression-like behaviors in female rats that experienced neonatal maternal separation. Psychoneuroendocrinology..

[54] Amodeo LR, Greenfield VY, Humphrey DE, Varela V, Pipkin JA, Eaton SE (2015). Effects of acute or repeated paroxetine and fluoxetine treatment on affective behavior in male and female adolescent rats. Psychopharmacology..

[55] Olivares-Nazario M, Fernández-Guasti A, Martínez-Mota L (2016). Age-related changes in the antidepressant-like effect of desipramine and fluoxetine in the rat forced-swim test. Behav Pharm.

[56] Fernández-Guasti A, Olivares-Nazario M, Reyes R, Martínez-Mota L (2017). Sex and age differences in the antidepressant-like effect of fluoxetine in the forced swim test. Pharm Biochem Behav.

[57] Levine S (2005). Developmental determinants of sensitivity and resistance to stress. Psychoneuroendocrinology..

[58] Marco EM, Llorente R, López-Gallardo M, Mela V, Llorente-Berzal Á, Prada C (2015). The maternal deprivation animal model revisited. Neurosci Biobehav Rev.

[59] McGrath JC, Lilley E (2015). Implementing guidelines on reporting research using animals (ARRIVE etc.): new requirements for publication in BJP. Br J Pharm.

[60] Kokras N, Antoniou K, Mikail HG, Kafetzopoulos V, Papadopoulou-Daifoti Z, Dalla C (2015). Forced swim test: What about females?. Neuropharmacology..

[61] Spear LP (2000). The adolescent brain and age-related behavioral manifestations. Neurosci Biobehav Rev.

[62] Slattery DA, Cryan JF (2012). Using the rat forced swim test to assess antidepressant-like activity in rodents. Nat Protoc.

[63] Bodnoff SR, Suranyi-Cadotte B, Aitken DH, Quirion AR, Meaney MJ (1988). The effects of chronic antidepressant treatment in an animal model of anxiety. Psychopharmacology..

[64] Blasco-Serra A, González-Soler EM, Cervera-Ferri A, Teruel-Martí V, Valverde-Navarro AA (2017). A standardization of the novelty-suppressed feeding test protocol in rats. Neurosci Lett.

[65] Turner CA, Gula EL, Taylor LP, Watson SJ, Akil H (2008). Antidepressant-like effects of intracerebroventricular FGF2 in rats. Brain Res.

[66] Slattery DA, Markou A, Cryan JF (2007). Evaluation of reward processes in an animal model of depression. Psychopharmacology..

[67] Unal G. Social isolation as a laboratory model of depression. Mental Health Effects of COVID-19. 2021; chapter 8:133–51.

[68] Curtis MJ, Alexander S, Cirino G, Docherty JR, George CH, Giembycz MA (2018). Experimental design and analysis and their reporting II: updated and simplified guidance for authors and peer reviewers. Br J Pharm.

[69] Michel MC, Murphy TJ, Motulsky HJ (2020). New author guidelines for displaying data and reporting data analysis and statistical methods in experimental biology. J Pharm Exp Ther.

[70] Schmidt MV, Wang XD, Meijer OC (2011). Early life stress paradigms in rodents: potential animal models of depression?. Psychopharmacology..

[71] Bis-Humbert C, García-Cabrerizo R, García-Fuster MJ (2021). Increased negative affect when combining early-life maternal deprivation with adolescent, but not adult, cocaine exposure in male rats: regulation of hippocampal FADD. Psychopharmacology..

[72] Bis-Humbert C, García-Fuster MJ (2021). Adolescent cocaine induced persistent negative affect in female rats exposed to early-life stress. Psychopharmacology..

[73] Armario A (2021). The forced swim test: historical, conceptual and methodological considerations and its relationship with individual behavioral traits. Neurosci Biobehav Rev.

