# Supplementary material for: Exploring pharmacological options for adolescent depression: a preclinical evaluation with a sex perspective
Source: Transl Psychiatry. 2022 Jun 1;12:220. doi: 10.1038/s41398-022-01994-y (PMC9160287; doi:10.1038/s41398-022-01994-y)
Supplement: Supplementary file 1 — Supplemental Materials [file 41398_2022_1994_MOESM1_ESM.doc]

**Supplemental Materials**

**Effects of early-life stress on affective-like behavior across time: effect of test repetition and/or age of testing**

The effects induced by early-life stress on the progression of affective-like behavior, as well as the impact of repeating behavioral tests across time, was evaluated in vehicle-treated rats (n=49 control and n=49 MD rats). As shown in Supplementary Fig. S2A for the time spent immobile (s) in the forced-swim test, a mixed-effects model analysis showed no significant effects of sex (F(1,94)=0.65, *p*=0.423) or early-life condition ((F(1,94)=0.64, *p*=0.426), but a significant effect of day (F(3.41,315.3)=32.47, *p*<0.001), with overall decreases in immobility from days 3-5 (adulthood) vs. days 1-2 (adolescence) of testing (up to an average drop of 36 seconds when comparing day 5 vs. day 1, ****p*<0.001). Similar results were observed for the time spent climbing (sex: F(1,94)=0.01, *p*=0.96, early-life condition: F(1,94)=0.68, *p*=0.413; day: F(3.34,308)=34.85, *p*<0.001), but with overall increases in from days 3-5 (adulthood) vs. days 1-2 (adolescence) of testing (up to an average raise of 37 seconds more climbing when comparing day 5 vs. day 1, ****p*<0.001). Interestingly, when evaluating swimming behavior, besides the significant effect of day (F(3.06,284.5)=9.14, *p*<0.001), and the lack of effect by early-life condition (F(1,94)=0.14, *p*=0.706), there was a significant effect of sex (F(1,94)=5.98, *p*=0.016), with female rats showing an increase in the time spent swimming (+4.0 ± 1.2 s vs. male rats, ****p*<0.001; see Supplementary Fig. S2A).

As for the data from the novelty-suppressed feeding test, when evaluating latency to center (see Supplementary Fig. S2B), there was a significant effect of sex (F(1,94)=26.19, *p*<0.001), and of day (F(1,90)=17.78, *p*<0.001), with male rats showing an overall higher latency to center as compared to female rats, which was also increased with repetition and/or age of testing (adulthood vs. adolescence for male controls rats, ****p*=0.0008, stats not shown in graph). However, there was no significant effect of early-life condition (F(1,94)=1.91, *p*=0.171). Similar statistical results were observed when evaluating feeding time (sex: F(1,94)=15.91, *p*<0.001; day: F(1,90)=41.41, *p*<0.001; early-life condition: F(1,94)=0.03, *p*=0.873) and distance travelled (sex: F(1,94)=44.10, *p*<0.001; day: F(1,88)=72.35, *p*<0.001; early-life condition: F(1,94)=1.04, *p*=0.310). For these outcomes, male rats showed lower feeding time (s) and distance travelled (cm) than their female counterparts, as well as decreased values with time (adulthood vs. adolescence).

**Supplementary Fig. S1 Monitoring the impact of early-life stress on affective-like behavior across time. A** Forced-swim test (FST) and **B** novelty-suppressed feeding test (NSF). Groups of treatment (only vehicle-treated rats): male-control (n=25), male-MD (n=25), female-control (n=24), female-MD (n=24). Data represents mean ± SEM of change in the particular behavioral traits evaluated (s or cm). Three-way ANOVAs or mixed-effects analysis (independent variables: sex, early-life and day of test) were performed.

**Table S1.** Three-way ANOVAs analyses for Fig. 2-5. Green-shadow boxes represent statistically significant comparisons.

**Table S2.** Two-way ANOVAs analyses (each sex separately) for Figs. 2-5. Green-shadow boxes represent statistically significant comparisons.

**Long-term effects of adolescent drug treatment in the forced-swim test**

The potential long-term effects of adolescent drug treatment (ketamine, cannabidiol, fluoxetine) were assessed in the forced-swim test during adulthood (45 days post-treatment). The results, as shown in Supplementary Fig. S2, described no overall changes in the behavioral traits analyzed (immobility, climbing or swimming), suggesting the antidepressant-like effects induced in adolescence return to normal over time.

**Supplementary Fig. S2 Long-term effects of adolescent drug treatment in the forced-swim in adult rats as measured 45 days post-treatment.** Data represents mean ± SEM of the time (s) spent immobile, climbing or swimming. Individual values are shown for each rat (symbols). Three-way ANOVAs (independent variables: sex, early-life and treatment) and two-way ANOVAs (independent variables: early-life and treatment) were performed with no significant differences observed (data not shown).
